# Nervonic acid and 15-epi-PGA1 mediate systemic mitochondrial dysfunction in AD dementia

**DOI:** 10.1007/s11357-025-01776-6

**Published:** 2025-07-11

**Authors:** Stephanie Robyn Heimler, K. Allison Amick, Jaclyn Bergstrom, Marcos Moliné, Gargi Mahapatra, Suzanne Craft, Anthony J. A. Molina

**Affiliations:** 1 Division of Geriatrics, Gerontology, and Palliative Care, Department of Medicine, University of California, San Diego, 9500 Gilman Drive, MC0665, La Jolla, CA 92093 USA; 2 Section On Gerontology and Geriatrics, Department of Internal Medicine, Wake Forest Baptist Medical Center, 1 Medical Center Blvd, Winston-Salem, NC 27157 USA; 3 Sam and Rose Stein Institute for Research On Aging, University of California, San Diego, 9500 Gilman Drive, La Jolla, CA 92093 USA

**Keywords:** Cognitive impairment, Alzheimer’s disease, Mitochondria, Bioenergetics, Circulating factors, Lipids

## Abstract

Systemic mitochondrial dysfunction is apparent in the pathophysiology of Alzheimer’s disease (AD). However, the factors driving bioenergetic decline remain unclear. This study utilized serum samples from older adults with normal cognition, mild cognitive impairment, and dementia to identify circulating molecules that can drive mitochondrial dysfunction in the context of AD. We used mass spectrometry to measure the abundance of lipid metabolites and applied tiered selection criteria to identify candidate “mito-inhibitory” molecules. These criteria were based on correlations with (1) in vitro bioenergetic effects of whole serum samples on naïve cells, (2) the bioenergetic capacity of blood cells from the serum donor, and (3) cognition, as measured by the modified mini-mental state exam. Mito-inhibitory lipid candidates were validated by examining their bioenergetic effects on neurons, myoblasts, and fibroblasts in vitro. Our results indicate that nervonic acid and 15-epi Prostaglandin A1 (15-epi-PGA1) are elevated in participants with dementia compared to those with normal cognition. Importantly, both metabolites inhibited mitochondrial function across multiple cell types in vitro. High resolution respirometric analyses reveal that inhibitory effects from lipid treatment occur via broad inhibition of the electron transfer system (ETS) with no change in overall mitochondrial content. This study provides insights into the mechanisms underlying systemic bioenergetic decline associated with AD dementia. The identification of circulating factors that drive mitochondrial bioenergetic decline may inform the development of mitochondrial therapeutics for AD.

**Graphical Abstract:**

In this study, we describe the identification and validation of mito-inhibitory bioactive lipid metabolites through a combination of serum lipidomics, statistical analyses, and mitochondrial respirometry.

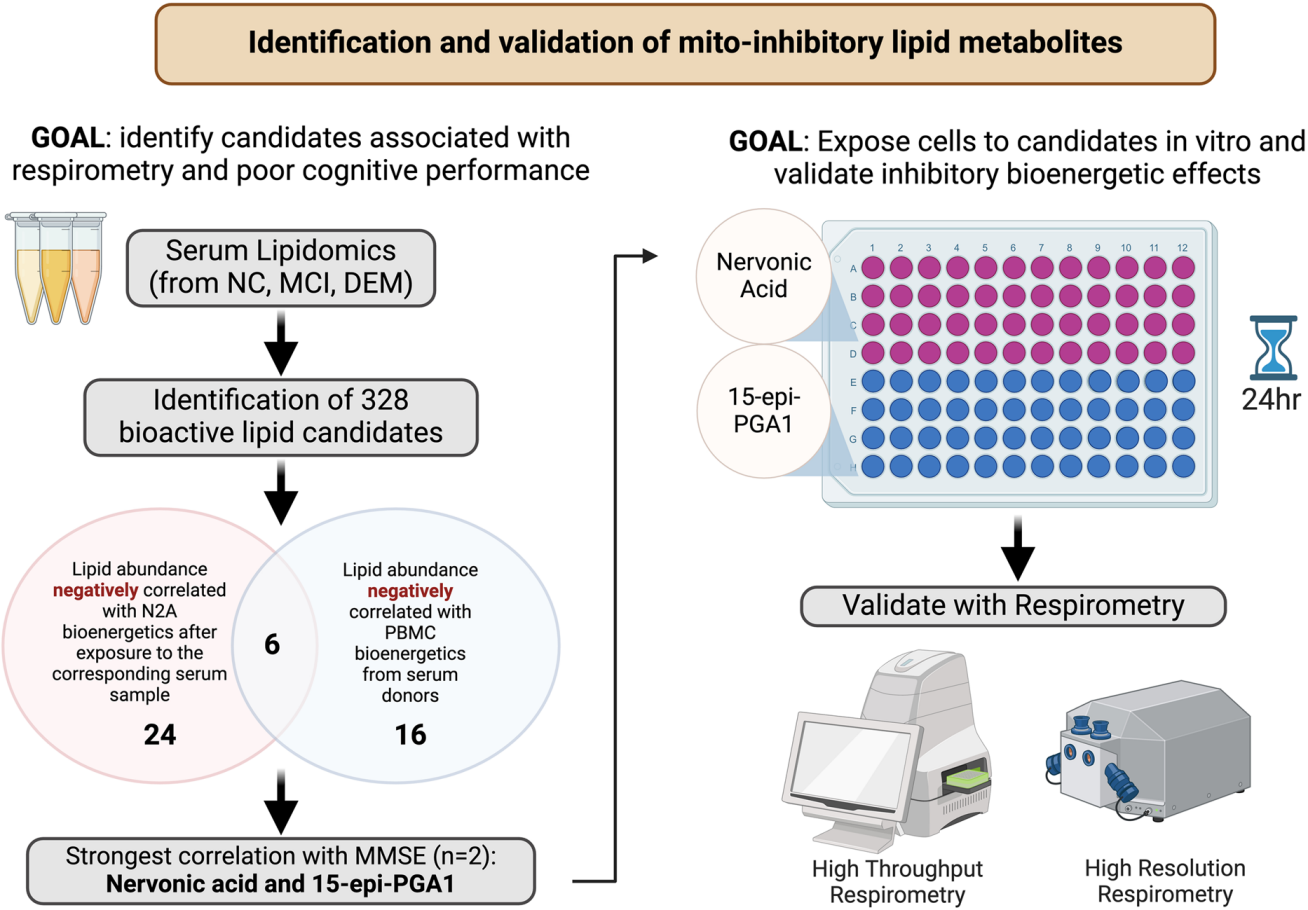

**Supplementary Information:**

The online version contains supplementary material available at 10.1007/s11357-025-01776-6.

## Introduction

Mitochondrial dysfunction associated with Alzheimer’s disease (AD) occurs in the brain and periphery. Mitochondrial alterations related to AD, such as decreased mitochondrial enzymatic activity, imbalance of mitochondrial fusion and fission, increased reactive oxygen species (ROS), impaired mitophagy, and bioenergetic dysfunction, have been documented in multiple neuronal and non-neuronal cell types [1–5]. Additionally, blood cells exhibit mitochondrial alterations associated with AD and dementia [6–8]. Our previous studies indicate that serum factors are associated with differences in mitochondrial function associated with age and cognition [9, 10]. We posit that circulating factors, such as hormones, cytokines, lipids, and other metabolites, underlie associations between systemic mitochondrial dysfunction and AD pathophysiology. In this project, we sought to identify specific circulating molecules that can drive systemic mitochondrial dysfunction and AD.

In this study, we focus on the effects of bioactive lipids on both neuronal and non-neuronal cells based on the recognition that AD and dementia-related pathologies exist both within and outside of the CNS [11, 12]. Bioactive lipids are involved in intercellular signaling and are associated with metabolic disorders, cardiovascular disease, inflammation, neurodegeneration, and AD [13–15]. The blood lipidome is distinct between people with AD compared to individuals with normal cognition. For example, differences in plasma bioactive lipid abundance are associated with genetic risk for AD from AD-related single nucleotide polymorphisms and polygenic risk scores of AD [16]. These studies implicate the dysregulation of sphingomyelins, phosphatidylcholines, phosphatidylethanolamines, and phosphatidylinositols in AD risk. Various plasma lipids are also associated with various AD biomarkers and AD clinical symptoms [17].

Based on the premise that factors in blood are associated with differences in mitochondrial function AD risk and progression, we previously investigated how naïve cells would respond to serum from donors with different cognitive statuses. We showed that neuronal cells exposed to serum from participants with normal cognition (NC), mild cognitive impairment (MCI), or dementia (DEM) exhibit differences in bioenergetic capacity associated with the cognitive ability of the serum donor [18]. Specifically, dilute serum from participants with MCI and DEM caused significant decreases in neuronal maximal respiration (Max) and spare respiratory capacity (SRC) compared to serum from participants with NC. To identify specific lipid metabolites underlying these in vitro bioenergetic effects, this previous study also performed targeted lipidomics and found that the abundance of lipid metabolites associated with inhibitory bioenergetic effects were correlated with worse cognitive function, whereas the abundance of lipid metabolites associated with positive bioenergetic effects were correlated with better cognitive function. While this study and others have linked electron transport chain (ETC) dysfunction to AD and dementia [19, 20], no previous study has reported a causal link between individual bioactive lipid metabolites and systemic ETC dysfunction associated with AD dementia.

The goal of this study is to identify bioactive lipids that can underlie systemic mitochondrial dysfunction associated with AD and dementia. Therefore, we focus on lipid metabolites with inhibitory effects on mitochondrial function (“mito-inhibitory”) and examine their direct effects on mitochondrial respiration as well as explore their mechanisms of action. This is the first study to examine the role of specific bioactive lipid metabolites in mitochondrial dysfunction in the context of AD dementia.

## Methods

### Participants

This study utilized the Wake Forest School of Medicine’s Alzheimer’s Disease Clinical Core (ADCC) cohort (IRB00025540; Title: Alzheimer’s Disease Clinical Core/“The Healthy Brain Study”; approval date: 1–8-2014/6–5-2024). The Wake Forest School of Medicine Institutional Review Board approved all procedures, and written informed consent was obtained from all participants and/or their legal representatives. Exclusion criteria, cognitive assessments, and clinical observations were conducted as previously described [18]. Serum samples from a subgroup of 59 participants (NC *n*: 20, MCI *n*: 21, and DEM *n*: 18) were used for this study.

### High-throughput respirometry

In order to deduce direct mitochondrial effects of serum or individual lipid metabolites, we performed high-throughput respirometry on N2a cells (CCL-131, ATCC), C2C12 cells (CRL-1772, ATCC), and primary human fibroblasts [21]. Respirometric analyses were conducted using an Agilent Seahorse XFe96 extracellular flux analyzer (XFe96; Agilent, Inc., Santa Clara, CA). The XF assay media contained XF DMEM Base Medium (Agilent Technologies, 103575–100) supplemented with 1 µM glucose (Agilent Technologies, 103577–100), 1 µM GlutaMAX (Thermo Scientific, 35050061), and 1 µM sodium pyruvate (Thermo Scientific, 11360070). The mitochondrial stress test Seahorse assay was conducted as previously described [18] with final in-well concentrations of 1.25 µM oligomycin, 0.5 µM (N2a), 0.75 µM (C2C12), or 1 µM (fibroblasts) FCCP, 1.0 µM Antimycin A, 1.0 µM Rotenone, and 32.4 µM Hoechst (nuclear DNA stain; 30-min incubation). The Seahorse analyzer is calibrated before each assay and is maintained regularly. Each assay was repeated two–three times, where the average and SEM are shown on relevant graphs. A representative trace of this assay is depicted in Supplemental Fig. 1A. Results were then normalized to imaging-based cell counts performed at the end of the assay which only capture adherent, live cells. These key steps to our approach support our ability to measure the mitochondria-specific effects of both lipid metabolites that are independent from potential effects on cell survival.

**Fig. 1:**
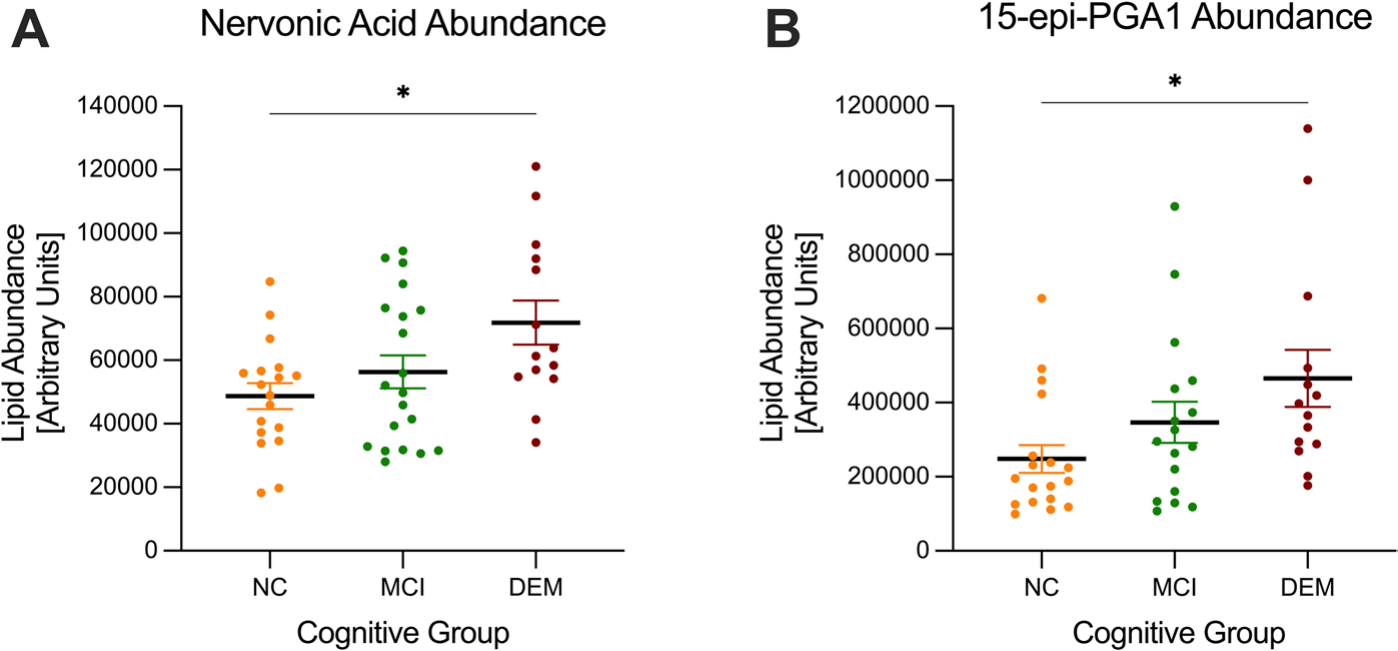
Lipid metabolite abundance in human serum from donors with NC, MCI, and DEM. Lipid abundance in human serum from donors with NC, MCI, and DEM. *P ≤ 0.05. Error bars are displayed as average $\pm$ SEM

### High-resolution respirometry

Peripheral blood mononuclear cell (PBMC) maximal uncoupled respiration (MaxETS) was measured by high-resolution respirometry (HRR) with an Oxygraph-2K (O2k; Oroboros Instruments, Innsbruck, Austria) in a previous study, and reaction conditions were performed as described [10] at 37 °C. Respirometric data from these assays with PBMCs were used in downstream screening and analysis. In order to investigate potential ETC entry-points impaired by nervonic acid and/or 15-epi-PGA1, we also conducted HRR in N2a cells to measure OXPHOS from fatty acid oxidation (FAO), complex I (CI), complex II (CII), maximal coupled respiration (MaxOXPHOS), MaxETS, and complex IV (CIV). After 24-hr treatment with lipid metabolites (explained below), cell culture flasks were washed with PBS to remove dead cells, and live cells were counted to ensure that only the live cells were loaded into the measurement chambers. This key step supports our ability to measure the mitochondria-specific effects of nervonic acid and 15-epi-PGA1 that are independent from potential effects on cell survival. Our protocol uses 450,000 N2a cells per 2-mL chamber. We used experimental concentrations of 2mM digitonin to permeabilize cell samples, followed by the injection of the following substrates: adenosine diphosphate (ADP; 0.5 M), magnesium (0.3 M), malate (0.1 M), octanoylcarnitine (0.1 M), cytochrome c (4 mM), malate (0.8 M), pyruvate (2 M), glutamate (2 M), succinate (1 M), and glycerol-3-phosphate (G3P; 1 M). The uncoupler, carbonyl cyanide-4 (trifluoromethoxy) phenylhydrazone (FCCP), is titrated in 1 mM steps until MaxETS is reached. Then, inhibitors of complexes I (rotenone, 1 mM) and III (antimycin-A, 2.5 mM) are added to halt mitochondrial respiration. After these inhibitors, ascorbate (As; 2 mM) and *N,N,N',N'*-tetramethyl-*p*-phenylenediamine dihydrochloride (TMPD; 0.5mM) are added. Ascorbate assists in keeping TMPD in a reduced state. At its reduced state, TMPD acts as an artificial substrate that reduces CIV. Because there is a finite amount of oxygen in the chambers, a reoxygenation step is necessary after the addition of ascorbate and TMPD. Finally, sodium azide (100 mM) is added as a CIV inhibitor. In order to measure OXPHOS at each entry point into the electron transfer system (ETS), FAO is measured after addition of fatty acid octanoylcarnitine. The breakdown of such fatty acids produces FADH2, which becomes a substrate for electron-transferring flavoprotein complex (CETF). FAO + CI respiration is measured after the addition of glutamate, as both glutamate and pyruvate contribute to the production of NADH, which is oxidized at CI. FAO + CI + CII respiration is measured after the addition of succinate, which is oxidized directly at CII. MaxOXPHOS is measured after the addition of G3P, which is metabolized by G3P dehydrogenase (G3P-DH). MaxETS is measured after addition of FCCP. CIV is measured as the difference between OXPHOS from ascorbate/TMPD and azide after reoxygenation. Each HRR assay was conducted with two–five biological replicates with two technical replicates each. The representative trace and data derived from these experiments are not corrected for residual oxygen consumption (ROX) with antimycin A. The oxygen fluxes are normalized to cell number. A representative trace of this assay is shown in Supplemental Fig. 1B.

### Cell culture and metabolite incubation

To validate lipid metabolites with predicted inhibitory action, we treated N2as, C2C12s, and primary human dermal fibroblasts with physiologically relevant concentrations of nervonic acid and 15-epi-PGA1, as would be present in human serum [22–24]. Although literature describing physiological concentrations of 15-epi-PGA1 was not available, the concentrations described in this work are based on optimizations with the physiological concentrations of the stereoisomer, PGA1 [22]. N2a, C2C12, and fibroblast cells treated with 8-iso-PGF1α were based on physiologically relevant concentrations in human seminal fluid [25], as concentrations in blood serum were unavailable. N2a and C2C12 cells were cultured in DMEM (Thermo Scientific, 11960044) supplemented with 10% FBS, 1% NEAA, 1% penicillin streptomycin, and 1% GlutaMAX. Fibroblasts were cultured in high glucose DMEM (Thermo Scientific, 1165092), supplemented with 15% FBS, 1% NEAA, and 1% GlutaMAX. Cells were plated in an Agilent Seahorse XFe96 assay plate at 10,000 cells/well (N2a and C2C12) or 20,000 cells/well (fibroblasts) in 160 µL culture media. Following cell seeding for both cell types, plates were allowed to rest for 45 min to 1 hr at room temperature before incubating at 37 °C and 5% CO_2_ for 24 hr ± 1 hr. Following the 24-hr incubation, culture media was removed from each well, and 160 µL of culture media containing each lipid metabolite concentration was added to each well in quadruplicate. Commercially available 15-epi-PGA1 (Cayman Chemical, 10070) was prepared in methyl acetate, and nervonic acid (Cayman Chemical, 13940) and 8-iso-PGF1α (Cayman Chemical, 15350) were prepared in DMSO. The use of vehicle controls enabled us to tease apart potential confounding effects. The same vehicle concentration (2.5%) was used for each condition, even as lipid metabolite concentrations were increased. This ensures that any dose-dependent decreases observed from nervonic acid and 15-epi-PGA1 are due to the effect of the lipid and not the vehicle.

All experiments were conducted between in vitro passage 4–16, and no morphological differences were observed between passages. Cell number per well was quantified using a Gen5 brightfield imager pre- and post-treatment, and these quantifications were confirmed by Hoechst nuclear stain post-treatment on the Seahorse XFe96 analyzer.

### Mitochondrial DNA quantification

In order to determine if differences cellular respiration were due to differences in mitochondrial content, we quantified the relative concentrations of mitochondrial DNA (mtDNA) to nuclear DNA (nDNA) using real-time quantitative PCR (rt-qPCR). First, cultured N2as were treated with either nervonic acid or 15-epi-PGA1 as well as their relevant solvent controls. After 24 hr of treatment, DNA from N2a cells was isolated and purified with the Gentra Puregene Cell Kit (Cat # 158745) using standard procedures. We prepared samples for DNA amplification with the PowerTrack™ SYBR™ Green Master Mix (Cat # A46012) using standard procedures. Five nanograms of DNA was used to amplify both mtDNA and nDNA genes. We amplified the mitochondrial gene, 16S, and the nuclear gene, β-actin, using the primer sequences listed in Table 1. Each primer was reconstituted at a stock concentration of 100 µM. The experimental concentration of each primer was 5 µM. qPCR was conducted using a QuantStudio3 machine (Thermo Fisher Scientific, Waltham, MA, USA), and was analyzed using Design and Analysis software (Thermo Fisher Scientific, Waltham, MA, USA). Each experiment was conducted with three–five biological replicates, and four technical replicates each. Each data point represents the average of the four technical replicates. DNA copy number (ΔCt) was used to compare mtDNA and nDNA expression in each group.

### Lipid metabolite abundance processing

Untargeted liquid chromatography-mass spectrometry (LC–MS) metabolomics was conducted on banked serum as previously described [18] to evaluate 328 bioactive lipid metabolite species in serum from the ADCC cohort. We shortened this catalog of all identified bioactive lipid metabolites to only those that could have predictive ability. We used a screening pipeline that integrates the abundance of various lipid metabolite species with participants’ bioenergetic and cognitive data to identify lipid species that inhibit mitochondrial function. First, arbitrary units for relative lipid abundance were quantified and processed through MetaboAnalyst (open source), a comprehensive platform for metabolomics data that processes, normalizes, and integrates multiple omics data. Quantification of relative abundance was conducted as follows: 17 lipid species of the total 328 were removed due to > 50% of the participants’ having missing values for those particular species. These species were likely missing due to being low-abundance lipid species that were below the limit of detection. If any participants had missing values for the remaining 311 lipid metabolites, these were estimated using feature-wise KNN (k-nearest neighbor) and were used in downstream analyses. Therefore, the final number of lipids analyzed was 311. We then compared lipid metabolite abundance to corresponding bioenergetic parameters from participants’ PBMCs as well serum-mediated bioenergetics from participants’ whole serum to predict those with direct inhibitory action in vitro.

### Statistical analysis

**Fig. 2:**
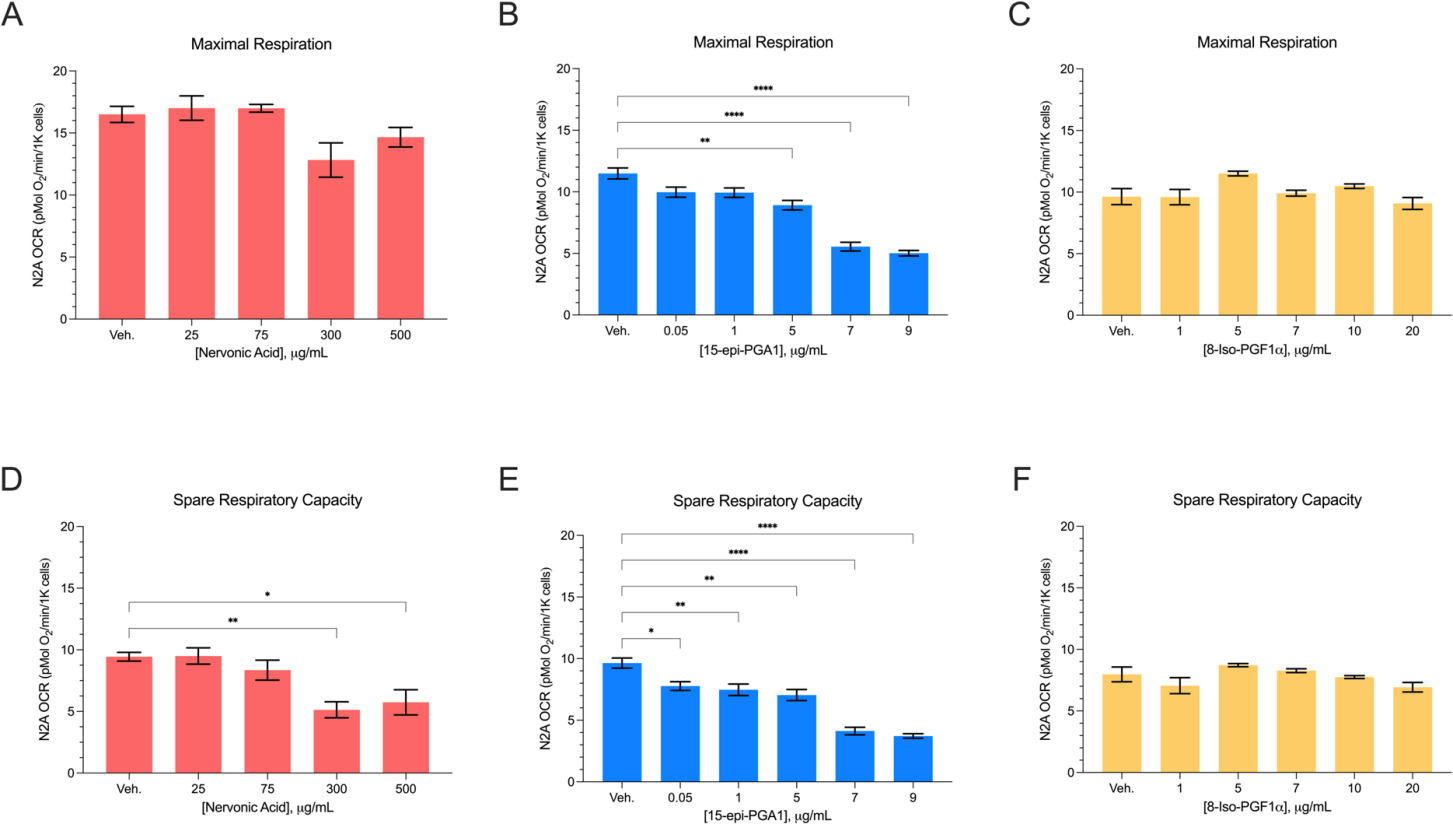
HTR of N2a cells after 24-hr treatments with mito-active lipids. N2a maximal respiration (**A**–**C**) and spare respiratory capacity (**D**–**F**) (pMol O_2_/min/1K cells) after 24-hr exposure to increasing doses of nervonic acid, 15-epi-PGA1, and 8-Iso-PGF1α. Data displayed are normalized to cell number. Vehicle control: 1.5% MeOAc or 2.5% DMSO. All error bars are displayed as average $\pm$ SEM. * *P* ≤ 0.05, ** ≤ 0.01, *** ≤ 0.001, **** ≤ 0.0001

**Fig. 3:**
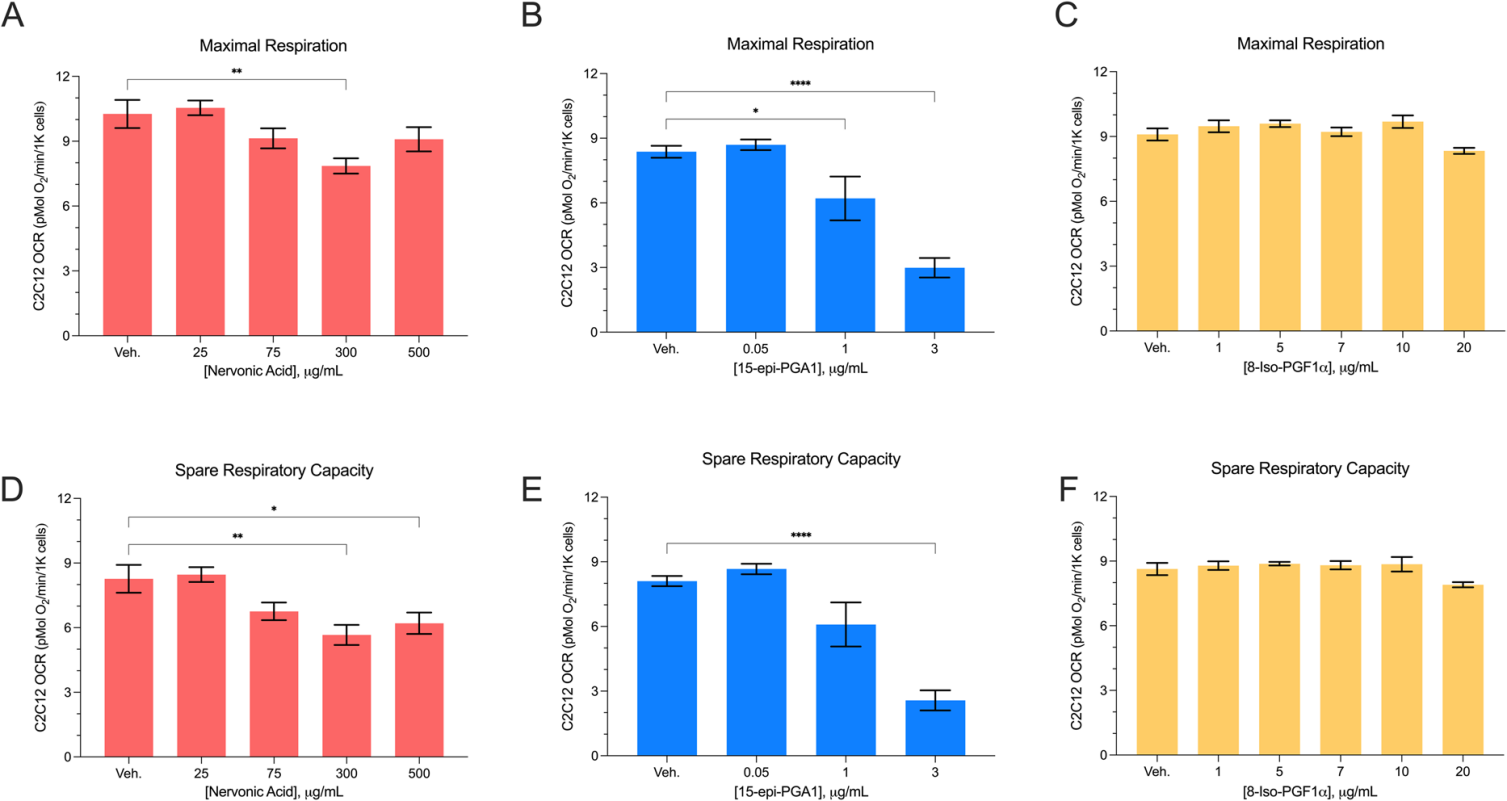
HTR of C2C12 cells after 24-hr treatments with mito-active lipids. C2C12 maximal respiration (**A**–**C**) and spare respiratory capacity (**D**–**F**) (pMol O_2_/min/1K cells) after 24-hr exposure to increasing doses of nervonic acid, 15-epi-PGA1, and 8-Iso-PGF1α. Data displayed are normalized to cell number. Vehicle control: 2.5% MeOAc or DMSO. All error bars are displayed as average $\pm$ SEM. * *P* ≤ 0.05, ** ≤ 0.01, *** ≤ 0.001, **** ≤ 0.0001

**Fig. 4:**
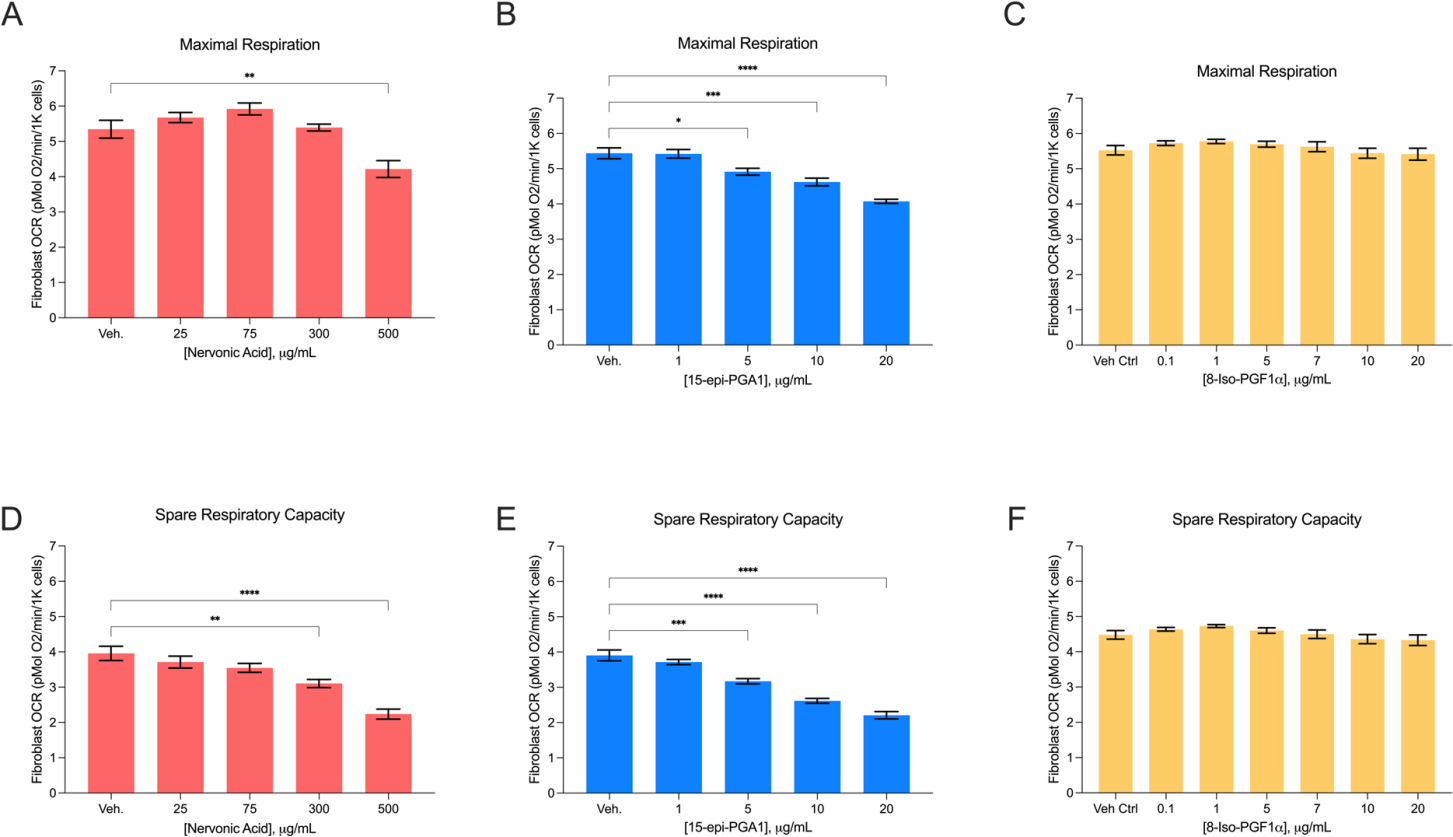
HTR of human fibroblasts after 24-hr treatments with mito-active lipids. Human fibroblast maximal respiration (**A**–**C**) and spare respiratory capacity (**D**–**F**) (pMol O_2_/min/1K cells) after 24-h exposure to increasing doses of nervonic acid, 15-epi-PGA1, and 8-Iso-PGF1α. Data displayed are normalized to cell number. Vehicle control: 2.5% MeOAc or DMSO. All error bars are displayed as average $\pm$ SEM. * *P* ≤ 0.05, ** ≤ 0.01, *** ≤ 0.001, **** ≤ 0.0001

**Fig. 5:**
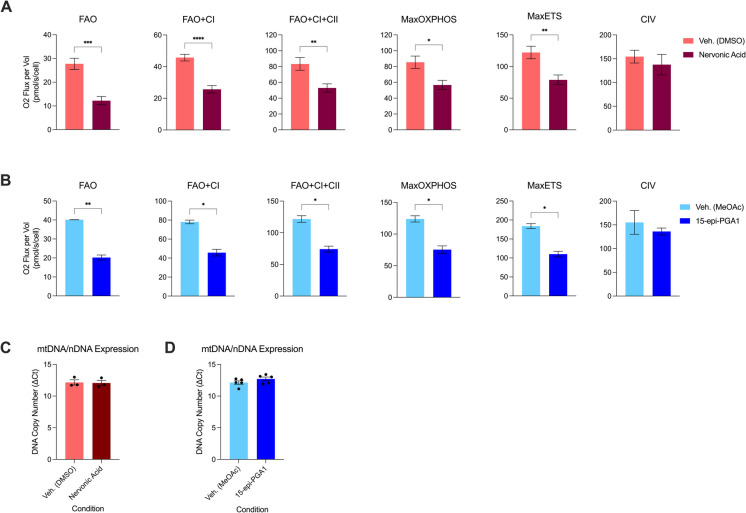
HRR and mitochondrial DNA expression of N2a cells treated with nervonic acid or 15-epi-PGA1. Differences in OXPHOS between 500 µg/mL nervonic acid and DMSO vehicle control (**A**) or 9 µg/mL 15-epi-PGA1 and MeOAc vehicle control (**B**) are shown. In A-B, OXPHOS mediated by FAO, FAO + CI, FAO + CI + CII, MaxOXPHOS, MaxETS, and CIV are measured. Treatment duration: 24 hrs. Vehicle control: 2.5% MeOAc or DMSO. N2a cell density: 450K/chamber. Oxygen fluxes are normalized to cell number. Mitochondrial DNA expression is quantified in cells treated with 500 µg/mL nervonic acid (**C**) or 9 µg/mL 15-epi-PGA1 (D)

Relationships between lipid abundance, N2a bioenergetics, PBMC bioenergetics, and cognitive measures (Graphical Abstract) were analyzed using Spearman correlations. Analyses of group differences between participant characteristics (Table 1 ), lipid metabolite abundance among cognitive groups (Fig. 1), and cell count data (Supplemental Fig. 3) were conducted with a one-way ANOVA test followed by a Dunnett’s post-hoc analysis for multiple comparisons. Analyses of dose–response histograms (Figs. 2, 3, and 4) were conducted using a one-way ANOVA test followed by a Dunnett’s post hoc analysis for multiple comparisons. Analyses comparing differences between treatment and control groups in HRR assays (Fig. 5) were conducted using a Student’s *t* test. All statistical analyses were performed using GraphPad Software, Inc. (Version 10.0.2; San Diego, CA).

**Table 1:** Primer sequences used for qPCR

Gene	Forward sequence	Reverse sequence
16S	GGAGCAATCCAGGTCGGTTTCTAT	GTCTTACCAATTTCCTGGCTCTG
β-actin	ACCTCCCTGAGTGTTTCTTGTG	GGTCCCGGCCAGCCAGGTCC

## Results

### Participant characteristics

Demographic and AD/dementia-related outcomes such as sex, race, apolipoprotein E (*APOE*; a gene that is a major genetic risk factor for AD), age, body mass index (BMI), hemoglobin A1C (HbA1c; measurement of the amount of blood glucose attached to hemoglobin in red blood cells), modified preclinical Alzheimer cognitive composite (mPACC5), white matter hyperintensities (WMH), hippocampal volume percentage (HV%), and cortical thickness were collected from the Wake Forest School of Medicine ADCC. These data are displayed in Table 2. There are no differences in participant sex, BMI, nor HbA1c among the three cognitive groups. Participants in the DEM group are slightly older on average (average age: 74.7 years), but there are no significant differences in age between NC and MCI groups (69.8 and 69.6 years, respectively). This cohort is comprised of primarily Caucasian participants (56/59 Caucasian, 3/59 Black or African American). The three human isoforms of *APOE*, *APOE2*, *APOE3*, and *APOE4* are the most prevalent genetic risk factors for AD [26, 27], and are quantified by cognitive group. mPACC5 scores were lower in participants with DEM compared to those with MCI or NC (− 5.139, − 0.949, 0.004, respectively; DEM vs. MCI: *P* < 0.0001, DEM vs. NC: *P* < 0.0001). WMH burden was greater in participants with DEM than those with MCI or NC (− 0.394, − 1.705, − 1.830, respectively; DEM vs. MCI: *P* < 0.0025, DEM vs. NC: *P* < 0.0009). HV% was lower in participants with DEM compared to those with MCI or NC (0.358, 0.477, 0.48, respectively; DEM vs. MCI: *P* < 0.0001, DEM vs. NC: *P* < 0.0001). Cortical thickness was lower in participants with DEM compared to those with MCI or NC (2.49, 2.65, 2.67, respectively; DEM vs. MCI: *P* < 0.0001, DEM vs. NC: *P* < 0.0001).

**Table 2:** Participant characteristics (*n* = 59)

	**NC (*n* = 20)**	**MCI (*n* = 21)**	**DEM (*n* = 18)**	
**Sex**				
**Female**	11	12	8	
**Male**	9	9	10	
**Race**				
**Caucasian**	20	20	16	
**Black/AA**	0	1	2	
**APOE**				
*ε4* +	8	6	9	
*ε4–*	12	14	9	
	**Avg. (SD)**			*** P * value**
**Age**	69.75 (6.82)	69.57 (5.75)	74.72 (6.85)	MCI vs. DEM: * P * = 0.042
**BMI**	24.95 (3.14)	26.59 (3.21)	27.34 (4.82)	NS
**HbA1c**	5.49 (0.3)	5.44 (0.2)	5.6 (0.55)	NS
**mPACC5**	0.004 (0.44)	− 0.95 (1.44)	− 5.14 (3.13)	NC vs. DEM, MCI vs. DEM: * P * < 0.0001
**WMH**	− 1.83 (0.88)	− 1.70 (1.38)	− 0.40 (0.98)	NC vs. DEM: * P* = 0.0009 MCI vs. DEM: * P *= 0.003
**HV%**	0.48 (0.06)	0.48 (0.07)	0.36, (0.08)	NC vs. DEM, MCI vs. DEM: * P * < 0.0001
**Cortical thickness**	2.67 (0.08)	2.65 (0.07)	2.49 (0.12)	NC vs. DEM, MCI vs. DEM: * P * < 0.0001

*APOE: *apolipoprotein E; *APOE ε4+: *APOE ε2/ε4, APOE ε3/ε4, APOE ε4/ε4. *APOE ε4–: *APOE ε2/ε2, APOE ε2/ε3, APOE ε3/ε3. One participant in the MCI group did not have APOE status recorded. *BMI: *body mass index. *HbA1c: *Hemoglobin A1c. For HbA1c parameter: NC, *n* = 8, *MCI* = 13, and *DEM* = 16. *AA: *African American. *mPACC5: *modified preclinical Alzheimer cognitive composite. *WMH: *white matter hyperintensities. *HV%: *hippocampal volume percentage

### Candidate identification

We analyzed the abundance of the 328 candidate lipid metabolites to identify those that inhibit mitochondrial function. Our first selection criterion identified metabolites whose abundances were negatively correlated with maximal respiration of naïve N2a cells treated with the corresponding ADCC serum sample, which was an indication of a potential mito-inhibitory effect. Our second selection criterion identified metabolites whose abundances were negatively correlated with the ADCC participants’ PBMC maximal respiration, which was an indication of a possible role in systemic mitochondrial dysfunction. PBMC maximal respiration and serum-mediated N2a maximal respiration based on the ADCC cohort were previously conducted in our laboratory and are available in published data sets [10, 18]. For each selection criterion, lipid candidates were considered inhibitory if the correlation coefficient between lipid abundance and bioenergetics (for N2a Max and PBMC MaxETS) was less than − 0.33 which corresponds to a *P* value less than 0.05. These criteria, which ranked lipid metabolite species by significance and strength of correlation, were used to identify lipid metabolites that would have potential meaningful effects on systemic mitochondrial function. Of the 328 lipid metabolites, 24 met the criteria for associating with N2a Max, and 16 met the criterion for associating with PBMC MaxETS. These shortlisted metabolites are displayed in Supplemental File 2. Of the lipid metabolites in these categories, six overlapped as having a negative correlation with both N2a Max and PBMC MaxETS with a correlation coefficient less than − 0.33 which corresponds to a *P* value less than 0.05. Of these six lipids, we identified two candidates whose abundance displayed the strongest negative correlation with mini mental state exam (MMSE) score (cognitive assessment of orientation, concentration, memory, and visuospatial skills). Based on these selection criteria, the two lipids with the strongest negative correlation with MMSE were identified as our primary molecules of interest: nervonic acid and 15-epi-PGA1. The six candidate inhibitory lipids based on these selection criteria are listed in Table 3, and our metabolite selection pipeline is described in the Graphical abstract.

### Nervonic acid and 15-epi-PGA1 are more abundant in serum from participants with dementia compared to those with normal cognition

To test the hypothesis that the abundance of nervonic acid and 15-epi-PGA1 in serum would increase with worse cognitive function, we compared levels of these two lipid metabolites among participants with NC, MCI, and DEM (Fig. 1). We found that the serum abundance of both nervonic acid and 15-epi-PGA1 is significantly elevated in participants with DEM compared to NC (47.6% elevated, *P* = 0.015 and 87.6% elevated, *P* = 0.026, respectively).

### Nervonic acid and 15-epi-PGA1 inhibit neuronal bioenergetic capacity

We validated the mito-inhibitory effects of nervonic acid and 15-epi-PGA1 by treating neuronal cells (N2a) with physiologically-relevant concentrations for 24 hrs in vitro. We observed a dose-dependent decrease in SRC, but not Max, in N2a cells treated with nervonic acid (Fig. 2A, D) and a decrease in both Max and SRC in N2a cells treated with 15-epi-PGA1 (Fig. 2B, E). We observed a reduction in SRC from 9.44 pmol O_2_/min in the vehicle control to 5.13 pmol O_2_/min (*P* = 0.005) and 5.74 pmol O_2_/min (*P* = 0.025) when N2as were exposed to 300 µg/mL and 500 µg/mL of nervonic acid, respectively. When N2a cells were exposed to 15-epi-PGA1 for 24 hr, cells exhibited a dose-dependent decrease in Max oxygen consumption rate (OCR) from 11.5 pmol O_2_/min in the vehicle control to 5.02 pmol O_2_/min when treated with 9 µg/mL (*P* ≤ 0.0001). We observed a similar dose-dependent decrease in SRC from 9.64 pmol O_2_/min in the vehicle control to 3.72 pmol O_2_/min after exposure to 9 µg/mL of 15-epi-PGA1 (*P* ≤ 0.0001). Bioenergetic data are normalized to cell number in Figs. 2, 3, 4, and 5. We observed lower cell numbers after treatment of N2a cells with increasing concentrations of nervonic acid, but there was no difference in cell number based on treatment concentration of 15-epi-PGA1 (Supplemental Fig. 3A, B).

As a control for the mito-inhibitory lipids, we used the same respirometric and metabolomics data sets to identify “mito-neutral” lipids that we predicted would not have an effect on mitochondrial function in vitro. Lipid metabolite candidates were considered to be likely mito-neutral if the Spearman correlation between lipid metabolite abundance and N2a Max, PBMC MaxETS, and MMSE was between − 0.1 and 0.1 with *P* > 0.5. Of the 328 lipid metabolites screened, 20 met these criteria. 8-iso-PGF1α was of particular interest as a mito-neutral control based on its weak correlation with N2a bioenergetics (*R* =  − 0.082, *P* = 0.55), PBMC MaxETS (*R* =  − 0.013, *P* = 0.86), and MMSE (*R* = 0.029, *P* = 0.68) as well as structural similarity to both 15-epi-PGA1 and nervonic acid. To our knowledge, no literature suggests that this metabolite is related to mitochondrial function nor AD dementia. When N2as were treated with a range of physiologically relevant concentrations of 8-iso-PGF1α, there were no differences in OCR compared to the vehicle control for Max nor SRC (Fig. 2C, F), and we did not observe differences in cell number with 8-iso-PGF1α treatment (Supplemental Fig. 3C).

**Table 3:** Circulating lipid metabolites associated with inhibition of mitochondrial respiration. *R* = Spearman correlation coefficient

Lipid Name	N2a Max (*R*, *P* value)	PBMC MaxETS (*R*, *P* value)	MMSE (*R*, *P* value)
FFA_Nervonic Acid [M-H + Acetate]	− 0.369, 0.005	− 0.399, 0.003	− 0.253, 0.053
EIC_15-epi-PGA1 [M-H]	− 0.433, 0.001	− 0.340, 0.014	− 0.204, 0.121
DOC_Resolvin D2 [M-H + Acetate]	− 0.503, < 0.001	− 0.356, 0.011	− 0.185, 0.169
EIC_161	− 0.488, < 0.001	− 0.429, 0.001	− 0.128, 0.336
FFA_Nervonic Acid [M-H]	− 0.369, 0.005	− 0.361, 0.009	− 0.167, 0.205
EIC_EPA [M-H + Acetate]	− 0.367, 0.015	− 0.365. 0.024	0.0298, 0.846

### 15-epi-PGA1 exerts a direct negative effect on myoblast bioenergetic capacity

AD-related mitochondrial dysfunction is observed in peripheral tissues such as skeletal muscle [28, 29]. We investigated if 15-epi-PGA1 and nervonic acid would have mito-inhibitory effects on myoblasts using similar physiologically relevant concentrations tested on N2a cells. We observed a decrease in Max when C2C12 cells were treated with 300 µg/mL nervonic acid compared to the vehicle control (7.86 pmol O_2_/min vs. 10.26 pmol O_2_/min, respectively;* P* = 0.006). We also observed a decrease in SRC, as C2C12 cells treated with 300 µg/mL and 500 µg/mL of nervonic acid exhibited lower OCR than the vehicle control (5.67 pmol O_2_/min, *P* = 0.002 and 6.21 pmol O_2_/min; *P* = 0.027, respectively vs. 8.27 pmol O_2_/min; Fig. 3A, D). There was no reduction in cell number when C2C12 cells were treated with nervonic acid (Supplemental Fig. 3D). There was a dose-dependent decrease in both Max and SRC when C2C12s were treated with 15-epi-PGA1 (Fig. 3B, E). C2C12 cells exhibited a reduction in Max from 8.38 pmol O_2_/min in the vehicle control to 6.21 pmol O_2_/min when treated with 1 µg/mL (*P* = 0.034) and 2.99 pmol O_2_/min when treated with 3 µg/mL (*P* ≤ 0.0001) of 15-epi-PGA1 for 24 hr. This result was similar for SRC, as 3 µg/mL of 15-epi-PGA1 reduced OCR from 8.11 pmol O_2_/min in the vehicle control to 2.57 pmol O_2_/min (*P* ≤ 0.0001). Concentrations of 5 µg/mL and higher of 15-epi-PGA1 on C2C12 cells resulted in a dramatic decrease in cell number and were therefore not used in this analysis (Supplemental Fig. 3E). Three milligrams per milliliter of 15-epi-PGA1 was found to be the highest usable concentration of this lipid metabolite on myoblast cells as higher concentrations were too toxic for the assays to be completed. When C2C12 cells were treated with the mito-neutral control, 8-iso-PGF1α, there were no differences in OCR for Max nor SRC compared to the vehicle control (Fig. 3C, F), nor were there any differences in cell number from treatment at any concentration tested (Supplemental Fig. 3F).

### Nervonic acid and 15-epi-PGA1 exert a direct negative effect on human fibroblast bioenergetic capacity

We investigated if 15-epi-PGA1 and nervonic acid have mito-inhibitory effects on human fibroblasts using similar physiologically relevant concentrations tested on N2a and C2C12 cells. We observed a decrease in Max when fibroblasts were treated with 500 µg/mL nervonic acid for 24 hrs compared to vehicle control (5.35 pmol O_2_/min vs. 4.22 pmol O_2_/min; *P* = 0.003; Fig. 4A). We observed a similar, but stronger dose-dependent decrease in SRC when fibroblasts were treated with both 300 µg/mL and 500 µg/mL, compared to the vehicle control (3.1 pmol O_2_/min, *P* = 0.003; 2.23 pmol O_2_/min, *P* ≤ 0.0001, respectively, vs. 3.96 pmol O_2_/min; Fig. 4D). There was a strong dose-dependent decrease in Max and SRC when fibroblasts were treated with 15-epi-PGA1 (Fig. 4B, E). Fibroblasts exhibited a reduction in Max from 5.44 pmol O_2_/min in the vehicle control to 4.07 pmol O_2_/min when treated with 20 µg/mL (*P* ≤ 0.0001) of 15-epi-PGA1 for 24 hr. This result was similar for SRC, as 20 µg/mL of 15-epi-PGA1 reduced OCR from 3.90 pmol O_2_/min in the vehicle control to 2.21 pmol O_2_/min (*P* ≤ 0.0001). When fibroblasts were treated with the neutral control, 8-iso-PGF1α, there were no differences in OCR for Max nor SRC compared to the vehicle control (Fig. 4C, F). We observed lower cell number in fibroblasts treated with nervonic acid, but not 15-epi-PGA1 nor 8-iso-PGF1α (Supplemental Fig. 3G-I).

### Bioenergetic effects of nervonic acid and 15-epi-PGA1 are mediated by broad inhibition of ETS activity

While we observed overall decreases in Max and SRC in multiple cell types (Figs. 2, 3, and 4), we sought to examine respiration driven by different entry points into the ETS: CETF, CI, CII, G3P-DH, and CIV. Analysis of respiration at these entry-points by HRR allows us to quantify OXPHOS in the following respiratory states: FAO, FAO + CI, FAO + CI + CII, MaxOXPHOS, MaxETS, and CIV (detailed explanation of quantification can be found in the “Methods” section). Figure 5A, B displays differences between lipid metabolite treatment and vehicle control in multiple respiratory states. Treatment of N2a cells with either 9 µg/mL 15-epi-PGA1 or 500 µg/mL nervonic acid, resulted in lower oxygen consumption across respiratory states. The only respiratory state which did not differ between treatment and control with both lipid metabolites was CIV-mediated OXPHOS. In order to deduce if decreases in mitochondrial activity at these respiratory states are mediated through a change in mitochondrial content, we measured the ratio of mitochondrial DNA expression (using the mitochondrial gene, 16S) to nuclear DNA expression (using µ-actin) after 24 hr of lipid or vehicle control treatment. Treatment of N2a cells with either 500 µg/mL nervonic acid or 9 µg/mL 15-epi-PGA1 (Fig. 5C, D) did not lead to differences in mitochondrial DNA expression, allowing us to conclude that decreases in OXPHOS at multiple respiratory states are due to direct impairments of the ETS, not due to changes in mitochondrial content.

## Discussion

This study is the first to identify and validate the mito-inhibitory effects of specific bioactive lipid metabolites associated with AD dementia. We combined lipidomics with HTR to identify a panel of candidate molecules that could directly inhibit mitochondrial function. The two inhibitory lipid metabolites, nervonic acid and 15-epi-PGA1, are elevated in serum from participants with dementia and are correlated with differences in brain morphology associated with AD dementia. These metabolites directly inhibit mitochondrial respiration in vitro across multiple cell types by broad inhibition of OXPHOS machinery.

Nervonic acid is a monounsaturated long-chain free fatty acid associated with brain development and injury repair [30]. Overall, the role of nervonic acid in the pathogenesis and progression of AD is not well understood, in part because studies suggest both detrimental and beneficial effects. It has been reported that levels of nervonic acid are increased in the brain tissue of AD patients, especially in the mid-frontal and temporal cortices, compared to healthy control patients [31]. In this same study, no difference was observed in the cerebellum, an area of the brain that is typically unaffected by AD. Further, a higher abundance of nervonic acid-containing sphingolipids in old mouse hippocampi suggests that this metabolite may contribute to age-related neurological dysfunction [32]. In contradicting studies, other groups have reported that nervonic acid can delay the decline of locomotion and learning ability in an AD mouse model [33]. While these reports differ on the effects of nervonic acid in the context of AD, it is possible that this lipid may have brain region-dependent effects. It is also possible that the level of nervonic acid in plasma or CSF changes long-term during AD pathogenesis and cognitive decline, causing it to have contrasting effects depending on when it is measured. Longitudinal studies investigating the long-term levels of nervonic acid during AD progression would be necessary to better understand its effects.

Prostaglandins have been associated with the exacerbation of neurodegeneration and neuroinflammation [14]. Studies have shown that treatment of PGA1 and prostaglandin E2 (PGE2) causes degeneration and decreased viability in neuronal cell lines with overexpressed amyloid precursor protein (APP) [34]. Clinically, PGE2 levels are increased in CNS tissue from patients with ALS [35] and Parkinson’s disease [36]. In this study, we identified the eicosanoid, 15-epi-PGA1, which is the 15(R) stereoisomer of PGA1 and was not previously described in the literature. This is the first study to show that 15-epi-PGA1 has a direct effect on mitochondrial function in vitro and is associated with AD.

A key feature of this study is the identification of mechanisms that nervonic acid and 15-epi-PGA use to exert OXPHOS impairments (Fig. 5). We found that both nervonic acid and 15-epi-PGA1 cause broad OXPHOS impairments across multiple respiratory states. Further, by observing that lipid metabolite treatment did not affect mitochondrial content, we provide evidence that the decreases in respiration are mediated primarily by ETS inhibition, rather than decreased mitochondrial content. Previous publications report that nervonic acid inhibits mitochondrial respiratory chain proteins cytochrome b (a key component within complex III) and NADH ubiquinone oxidoreductase subunit B8 (NDFUB8; a key component within complex I) to activate inflammatory pathways [37]. Prostaglandins have been shown to cause oxidative stress associated with reduced cell viability [38, 39] as well as with activation of PINK–Parkin mitophagy [40]. Prostaglandins may also impair the ubiquitin–proteasome pathway in the context of neurodegeneration [41]. While prostaglandins have diverse roles and mechanisms of action, similar studies of the bioenergetic effects of 15-epi-PGA1 are lacking, and it is possible that it may cause broad ETS disruption through similar or different pathways. In line with these findings, a multitude of ETC dysfunctions have been previously reported in AD. Studies have indicated both broad ETC dysfunction [42, 43] as well as impairments in specific complexes or entry-points in the ETC. For example, APP/PS1 mouse models have indicated specific decreases in CII activity [20]. Studies have also shown CIV impairments in both the brain and periphery in AD [44–46], although, interestingly, we did not observe differences in CIV respiration when comparing mito-inhibitory lipid treatments to vehicle controls. These data suggest that while nervonic acid and 15-epi-PGA1 may impair CETF, CI, CII, and G3P-DH, which all donate electrons to the ETS, they may not impair CIV, the final site of oxygen consumption. This, in turn, may have implications in maintaining ATP production.

Our data indicates that treatment with nervonic has differential effects on maximal uncoupled respiration when measured in intact cells using the Seahorse assay versus permeabilized cells using the O2k (Figs. 2A and 5A). It should be noted that different protocols are used to measured maximal uncoupled respiration in HRR compared to HTR. The HRR protocol titrates the addition of FCCP, ensuring that maximal uncoupled respiration is achieved; therefore, we believe that nervonic acid leads to a shift in the FCCP response and decrease in maximal respiration. We observed that high concentrations of nervonic acid were cytotoxic to both N2a cells and fibroblasts, but not to C2C12 cells (Supplemental Fig. 3A,D,G). We also observed that 15-epi-PGA1 was cytotoxic to C2C12 cells, but not to N2a cells nor fibroblasts (Supplemental Fig. 3B,E,H). As expected, the mito-neutral control lipid metabolite, 8-iso-PGF1α, did not decrease cell number in any cell type for any concentration tested (Supplemental Fig. 3C,F,I). While both inhibit mitochondrial function, 15-epi-PGA1 had a stronger effect on C2C12 cells than fibroblasts or N2as, while nervonic acid had a stronger effect on fibroblasts and N2as than C2C12 cells for reasons still unknown. While we anticipate that different mechanisms can underlie differences in sensitivities of various cell types to nervonic acid and 15-epi-PGA1, inherent differences in metabolic demand and capacity are likely contributors. It is expected that mito-inhibitory molecules can be cytotoxic at high concentrations, as any compound that is toxic to mitochondria (“mito-toxic”) will eventually be toxic to the cell. While we observed differences in cell death at the highest concentration of lipid treatments, it should be noted that the range of concentrations tested were based on physiologically relevant concentrations found in vivo. Moreover, the concentration of the vehicle remained the same in every condition, ensuring that the vehicle itself did not have toxic effects on cells. Importantly, all respirometric data reported in this manuscript were normalized to total number of live cells for the HTR and HRR assays. For the HRR assay which was conducted at only the highest concentrations of nervonic acid and 15-epi-PGA1, the wash steps after treatment with each lipid helps to ensure that primarily live cells are loaded into the measurement chambers. For the HTR assays, which were conducted at every concentration of lipid metabolite, the results were normalized to imaging-based cell counts performed at the end of the assay that only capture adherent, live cells. Therefore, the mitochondrial effects of both nervonic acid and 15-epi-PGA1 reported here are primary to effects on cell survival.

In this study, we focused on the effects of nervonic acid and 15-epi-PGA1 in both neuronal and non-neuronal cells due to mounting evidence that AD and dementia-related pathologies exist in the CNS and periphery. As such, multiple lines of evidence indicate robust brain-body axis connections, as both physical activity and diet have strong implications on cognition and neurodegenerative disease. For example, previous findings indicate that exercise can improve cognitive function and hippocampal plasticity in mice, especially in APOEε4 carriers who are at genetically higher risk of AD [47], highlighting the importance of this brain-body connection. Animal models have also shown that caloric restriction can improve learning and memory as well as decrease oxidative stress [48]. Along these same lines, nervonic acid can be found in some food sources, such as fish oils and seed oils of the *Lunaria annua* plant [49], potentially providing an explanation for how plasma nervonic acid levels may be affected by diet. We focus on three different cell types in this study (neurons, myoblasts, and fibroblasts) to establish how both central and peripheral cell types can respond to systemic factors in serum. Our data suggest that mito-inhibitory molecules can directly impact multiple cell types representing the CNS and the periphery. We identified nervonic acid and 15-epi-PGA1 in serum, and therefore believe that these circulating lipid metabolites may have the ability to elicit responses systemically across all cell types with which they come into contact. For this reason, we are interested in the effect of these lipid metabolites in myoblasts, fibroblasts, and neurons. Since the myoblasts and neurons were derived from animal models, we included primary human fibroblasts in our testing to support the translatability of our findings. Further, there is mounting evidence that these three cell types are all sensitive to AD-related mitochondrial dysfunction. In neurons, a wide array of mitochondrial dysfunction can be observed from reduction of cytochrome oxidase activity to abnormal mitochondrial dynamics to impaired glucose metabolism [1, 2, 4]. In skeletal muscle, it has been found that higher in vivo bioenergetic capacity is associated with lower risk of developing AD/dementia through cognitive testing and AD biomarkers [50]. It has also been shown in AD models that skeletal muscle cells have lower oxygen consumption rates as well as alterations to mitochondrial membrane compositions [28, 51]. Much work has also been conducted to show that skeletal muscle mitochondrial function is associated with physical and cognitive abilities [52–55], both of which decline with AD. Such manifestations are also observed in fibroblasts, such as impaired metabolic potential, redox potential, and mitophagy [3, 5]. Therefore, these three cell types are all vulnerable to changes in mitochondrial dysfunction, especially in the case of AD.

This study was designed primarily to identify drivers of mitochondrial function in the context of AD and shed light on the mechanism by which mitochondrial dysfunction might arise. It may be noted that nervonic acid and 15-epi-PGA1 may also have diagnostic applications and can be evaluated along with established AD biomarkers in larger cohort studies*.* Future longitudinal studies in humans may help identify if changes in nervonic acid and 15-epi-PGA1 over time correspond with changes in cognition and mitochondrial bioenergetics. It also remains to be established if nervonic acid and 15-epi-PGA1 can cross the blood–brain barrier (BBB) in the context of AD. It has been shown that nervonic acid can cross the BBB in newborns [56], but this has not yet been shown in adults. However, many free fatty acids (i.e., nervonic acid) should be able to cross the BBB through various transporters, such as major facilitator superfamily domain-containing protein 2 [57]. Prostaglandins can also cross the BBB, but the specific mechanism of 15-epi-PGA1 transport through the BBB is still unclear. Future studies are also needed to establish causality between the abundance of nervonic acid and 15-epi-PGA1 and cognitive decline in AD. Such studies may investigate the effects of these lipid metabolites in animal models. We recognize that there are a number of AD transgenic mouse models available. These model different types of AD, such as sporadic AD (SAD) or familial AD (FAD). Future experiments designed to investigate the casual effects of nervonic acid and 15-epi-PGA1 may initially focus on SAD, where there is a need to understand molecular underpinnings of systemic bioenergetic decline, but can also be expanded to models of FAD, which may include common AD mutations in amyloid precursor protein (APP), presenilin 1 (PSEN1), or presenilin 2 (PSEN2) [58, 59]. In animal models, it would be informative to infuse lipid metabolites at physiological and pathological levels and then examine resulting bioenergetic effects in a multitude of tissues in the CNS and periphery. It would also be important to investigate the lipid metabolites’ causal effects on AD pathology, such as on cognition, brain histology, and levels of blood and CSF biomarkers. Lastly, the approaches described in this study may also be adopted for other biorepositories and to support the discovery of other factors driving systemic mitochondrial bioenergetic decline.

## Supplementary Information

Below is the link to the electronic supplementary material.

ESM 1 (PDF 611 KB)
