# Supplementary material for: Nervonic acid and 15-epi-PGA1 mediate systemic mitochondrial dysfunction in AD dementia
Source: GeroScience. 2025 Jul 11;48(2):2327–43. doi: 10.1007/s11357-025-01776-6 (PMC12972455; doi:10.1007/s11357-025-01776-6)

Supplemental Figure 1: **Representative Seahorse and O2K traces.** A) Representative Seahorse trace depicting one assay with N2a cells treated with a DMSO or various concentrations of nervonic acid. B) Representative O2k trace with substrates, uncouplers, and inhibitors, as described in methods.

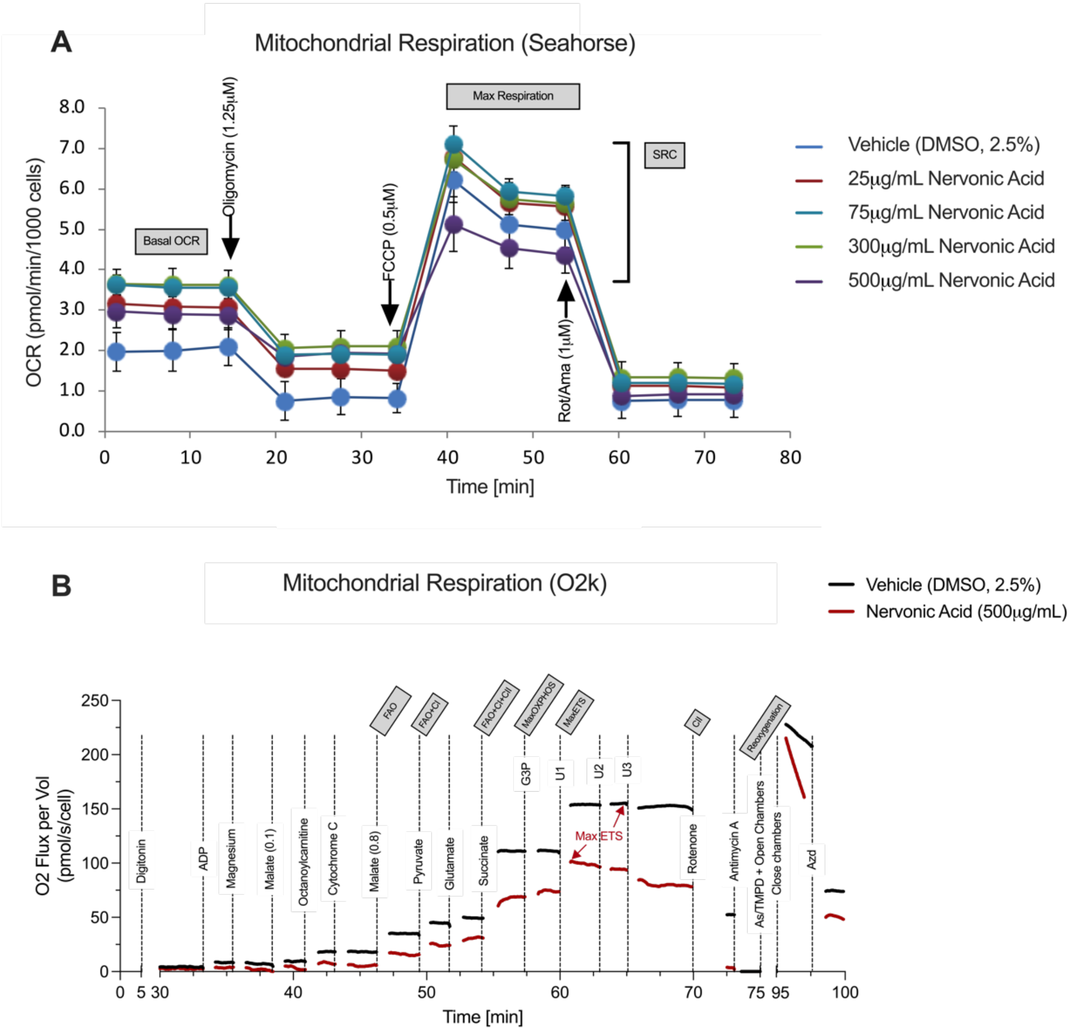

Supplemental File 2: Shortlisted Lipids from identification pipeline. Please see supplemental file (excel sheet) with shortlisted lipids at each step.

Supplemental Figure 3: **Cell count 24hr after lipid metabolite treatment.** A-C) Cell counts after 24hr treatment with nervonic acid, 15-epi-PGA1, and 8-iso-PGF1α for N2as (A-C), C2C12s (D-F), or fibroblasts (G-I).

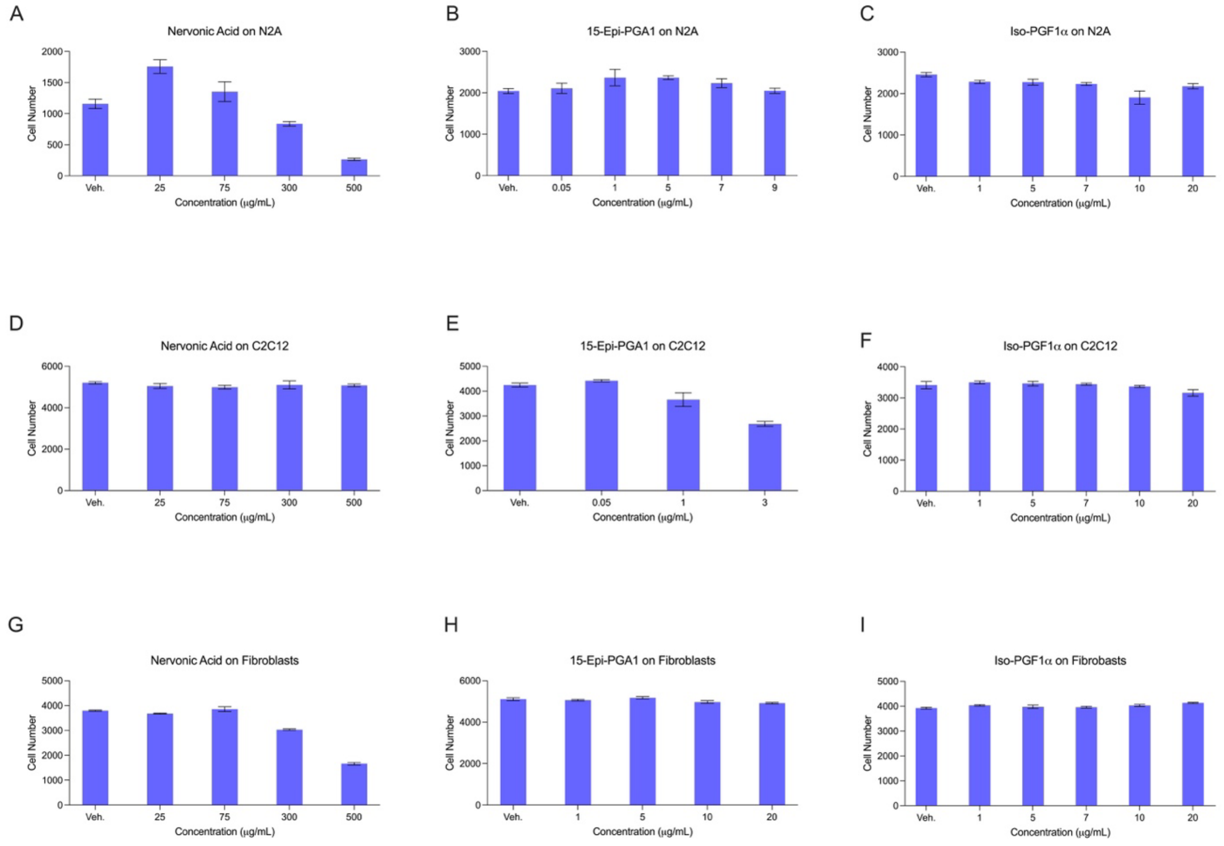

Supplement: Supplementary file 1 — (PDF 611 KB) [file 11357_2025_1776_MOESM1_ESM.pdf]
